# Vine-inspired zinc-ion modified black phosphorus coating accelerates bone tissue infiltration of 3D printed scaffolds

**DOI:** 10.7150/thno.113623

**Published:** 2025-04-09

**Authors:** Dan Li, Danni Dai, Jianrong Wang, Yan Wang, Yujia Tian, Chao Zhang

**Affiliations:** Stomatological Hospital, School of Stomatology, Southern Medical University, Guangzhou 510280, China.

**Keywords:** diabetic bone defect, osteogenesis-angiogenesis coupling, mitochondrial dynamics, bio-inspired, 3D printed scaffold

## Abstract

**Rationale:** In the reconstruction of diabetic bone defects, 3D-printed scaffolds often encounter the challenge of limited and delayed tissue ingrowth in their central regions, which is critical for successful osseointegration and prognostic outcomes. Hyperglycemia induces endothelial apoptosis and impedes angiogenesis, thus inhibiting osteogenic differentiation of bone marrow stem cells (BMSCs).

**Methods:** Drawing inspiration from the growth pattern of vines, we developed a Zn@BP/Si coating on the 3D-printed titanium scaffold to promote the coupling of angiogenesis and osteogenesis. This coating was achieved by Zn^2+^-modified black phosphorus (BP), which not only enhances the stability and photothermal properties of BP, but also prevents endothelial apoptosis. The effectiveness of Zn@BP/Si in the reconstruction of diabetic bone defects was investigated in rat model of diabetic femoral defect. Its effect on osteogenesis-angiogenesis coupling has also been explored in BMSCs and HUVECs.

**Results:** Zn@BP/Si regulated mitochondrial dynamics and provided motivation for cell adhesion and migration, just like the climbing of vines. Notably, the regulation of enzymatic activity plays a crucial role in its inhibition of excessive mitochondrial fission. The results demonstrate that the Zn@BP/Si promotes the growth of “vascular vines” and ameliorates the angiogenic and osteogenic inhibition in diabetes.

**Conclusions:** The study reveals the potential of bio-inspired Zn@BP/Si coating in angiogenesis-osteogenesis coupling and the treatment of diabetic bone defects.

## Introduction

Hyperglycemia degrades the density, morphology, and turnover of bone tissue, and thus predisposes diabetic patients to osteoporosis, fractures, and delayed bone healing. Over the past two decades, the global incidence of diabetes has been rising steadily, while life expectancy has been prolonged significantly. Consequently, diabetic osteopathy has emerged as a significant public health concern 1. In the reconstruction of diabetic bone defects, 3D-printed scaffolds often encounter the challenge of limited and delayed tissue ingrowth in their central regions, which is critical for successful osseointegration and prognostic outcomes. Under homeostatic conditions, endothelial and mesenchymal stem cells in the bone marrow and peripheral blood migrate to bone lesions, proliferate, differentiate, and promote angiogenesis and osteogenesis. However, under high-glucose conditions, accumulation of advanced glycation end products causes oxidative stress and thereby impairs the activation, adhesion, proliferation, and angiogenic capabilities of endothelial cells and bone marrow stem cells (BMSCs). The subsequent impairment of angiogenesis and osteoblastic function predisposes to fractures and delayed bone healing 2, consequently exacerbating both the suffering of diabetic patients and associated economic burdens 3, 4. Osteogenesis-angiogenesis coupling, essential for bone repair, is disrupted in diabetes through the accumulation of advanced glycation end products. Enhancing the secretion of osteogenic factors like VEGF and PDGF-BB by endothelial cells can promote the osteogenic differentiation of BMSCs into osteoblasts, thereby restoring the healing of bone defects.

Mitochondria comprise dynamic, interconnected organelle networks that maintain the balance of their quantity, quality, and function through continuous fission, fusion, and transfer. The breakdown of the mitochondrial dynamic balance by sustained hyperglycemia represents an upstream event leading to diabetic nephropathy, microvascular disease, and metabolic osseous complications 5. Drp1 expression, which regulates mitochondrial fission, is upregulated under hyperglycemic induction, leading to mitochondrial fragmentation and dynamic imbalance. Mitochondrial-targeted antioxidants can significantly inhibit bone loss in diabetic or high fat diet-treated mice 6. Inhibition of mitochondrial fission in the hyperglycemic environment before ischemia-reperfusion can normalize Ca^2+^ balance, reduce ROS generation, recover mitochondrial membrane potentials in the cardiomyocytes of diabetic rats, and reduce cardiac sensitivity to ischemia-reperfusion 7. Therefore, drugs or biomaterials that regulate mitochondrial biogenesis, energy metabolism, or quality control may potentially mitigate hyperglycemia-induced dysfunction. This approach represents a major new strategy for restoring cellular function 8. Current attention is focused primarily on mitochondria in skeletal muscle and liver, which may have the greatest impact on glucose homeostasis as potential targets for glucose regulation. However, studies on regulating endothelial mitochondria to promote bone regeneration are limited.

**Scheme 1:**
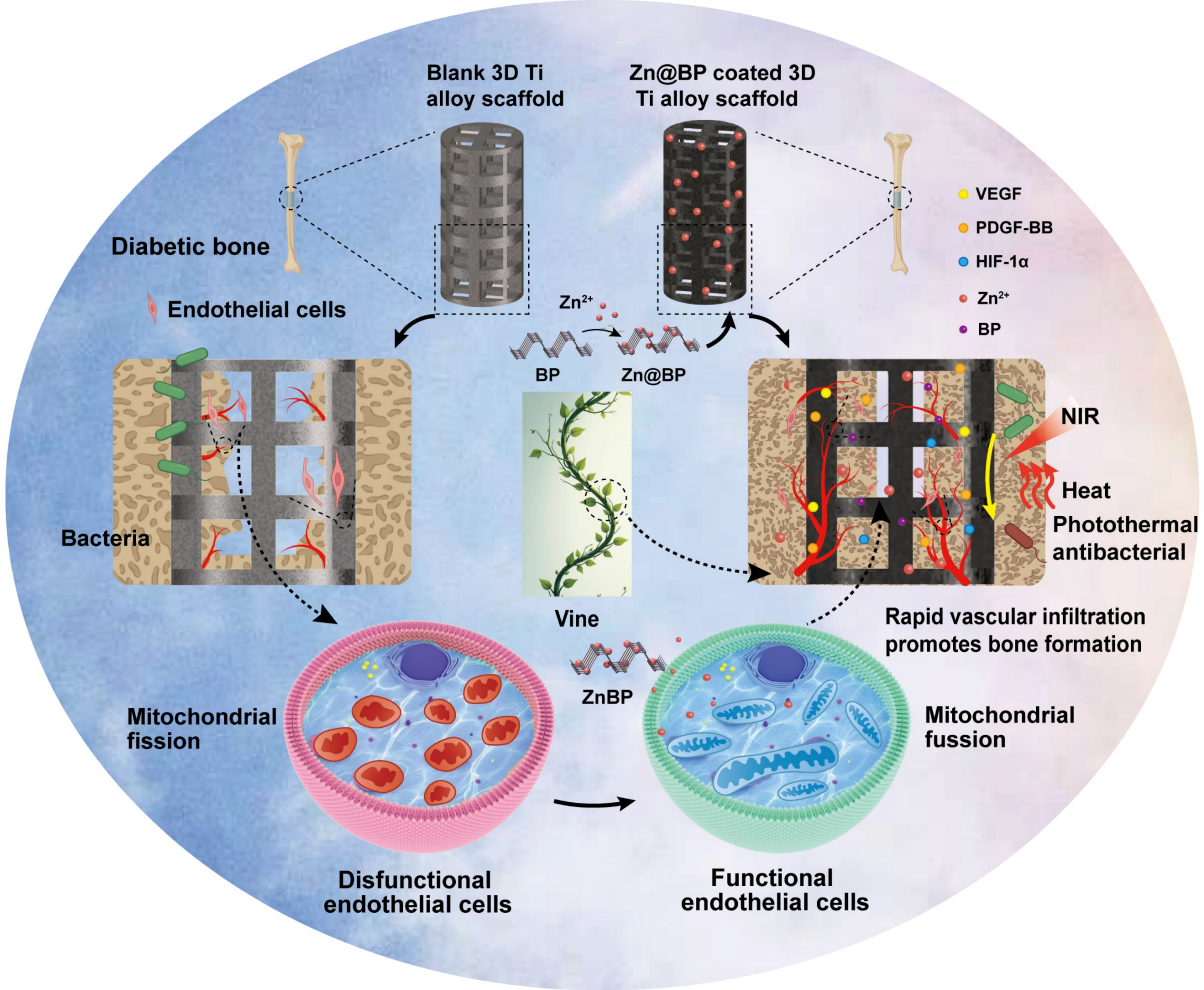
Construction of Zn@BP coated 3D-printed titanium alloy scaffold and its photothermal antibacterial performance and mechanism of angiogenesis-osteogenesis coupling to promote osteogenesis in diabetes.

Drawing inspiration from the growth pattern of vines, we developed a Zn@BP/Si coating on the 3D-printed titanium scaffold to regulate endothelial mitochondria and promote the coupling of angiogenesis and osteogenesis. (**Scheme 1**). Black phosphorus (BP) is a novel nanomaterial discovered in the past decade. It is composed solely of phosphorus with a large specific surface and may be functionalized easily. BP nanosheets can degrade *in vivo* to generate phosphate ions, which capture Ca^2+^ to form calcium phosphate and induce bone mineralization. BP has photothermal conversion properties that generate heat and kill bacteria in response to near-infrared (NIR) light without promoting drug resistance. Our previous studies have confirmed that BP-containing coatings on implant surfaces exert anti-infective and pro-healing activities. However, the lone pairs of electrons on phosphorus atoms can easily react with oxygen or water to form P-O bonds, which enable material degradation and, eventually, loss of its biological properties. Metal ions with vacant orbitals can coordinate with the lone pairs of electrons on BP and achieve highly stable microstructures and properties. Consequently, we introduced Zn^2+^ to modify the surface of BP nanosheets and utilized silanization to fix Zn^2+^-modified BP onto the surface of 3D-printed titanium alloy scaffolds. It is supposed to provide motivation for cell adhesion and migration, just like the climbing of vines. The restoration of mitochondria provides the power for blood vessels to climb on the surface of the scaffold, thereby not only preventing infections, but also enhancing bone repair. Here, the effects of Zn@BP on bone defect repair were evaluated in diabetes, with particular attention being given to the role of the angiogenesis-osteogenesis coupling and the impact of this process on intracellular mitochondrial dynamics.

## Results and Discussion

### Construction and characterization of 3D-printed Ti scaffolds with silanated Zn@BP coating

**Figure 1:**
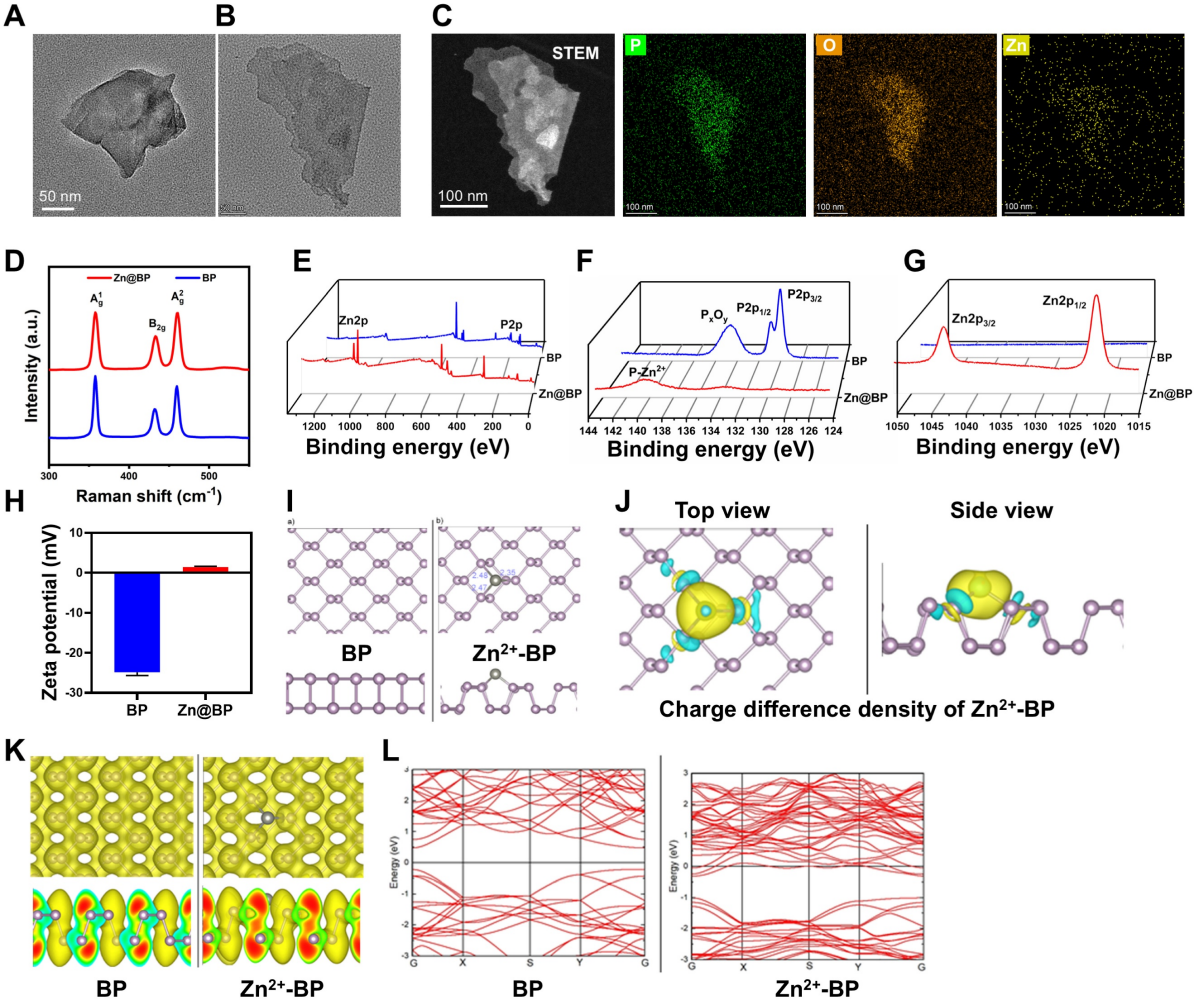
Construction and characterization of Zn@BP/Si nanosheets. (A)TEM assessment of BP nanosheets. (B) TEM assessment of Zn@BP/Si nanosheets. (C) STEM-EDX elemental mapping of Zn@BP/Si nanosheets. (D) Raman spectra of BP and Zn@BP/Si nanosheets. (E-G) XPS data of BP and Zn@BP/Si nanosheets. (H) Zeta potentials of BP and Zn@BP/Si nanosheets. (I) Optimized structures of P and Zn^2+^-BP, the selected bond lengths are in Å. (J) Calculated charge difference distribution of Zn^2+^-BP. (K) Calculated electron localization function (ELF) maps of P and Zn^2+^-BP. (L) Calculated energy band structures of P and Zn^2+^-P.

Transmission electron microscopy (TEM) showed that the average size of BP and Zn@BP nanosheets was approximately 200 nm, with sharp edges (**Figures 1A-B**). Energy dispersive X-ray spectroscopic (EDS) mapping of Zn@BP revealed that phosphorus (P), oxygen (O), and zinc (Zn) were distributed uniformly throughout the nanosheet (**Figure 1C**). Raman spectra showed that the characteristic peaks of A^1^_g_, B_2g_, and A^2^_g_ of BP nanosheets were retained after Zn^2+^ modification (**Figure 1D**). X-ray photoelectron spectroscopy (XPS) showed the Zn2p peak appearing at 1022 eV, confirming the modification of BP by Zn^2+^ (**Figures 1E-G**). The zeta potential of Zn@BP increased to approximately +1.41 mV (**Figure 1H**). This increase is due to neutralization of the negative charge by divalent zinc ions, with the resulting metal phosphides constituting a protective layer that enhances the stability of BP. Theoretical calculations indicated that the binding energy of Zn^2+^ on the BP monolayer was -7.6 eV at the hollow site, indicating that Zn^2+^ can bind stably to the surface, forming strong coordination bonds with three phosphorus atoms (**Figure 1I**). The calculated charge difference density and electron localization function further demonstrated that electron transfer occurs between Zn^2+^ and P, resulting in strong metal-coordination bonds (**Figures 1J-K**). The adsorption of Zn^2+^ reduced the bandgap of BP from 0.69 eV to 0.35 eV, suggesting that electrons in the valence band are more easily photoexcited, thus enhancing photothermal conversion performance (**Figure 1L**).

**Figure 2:**
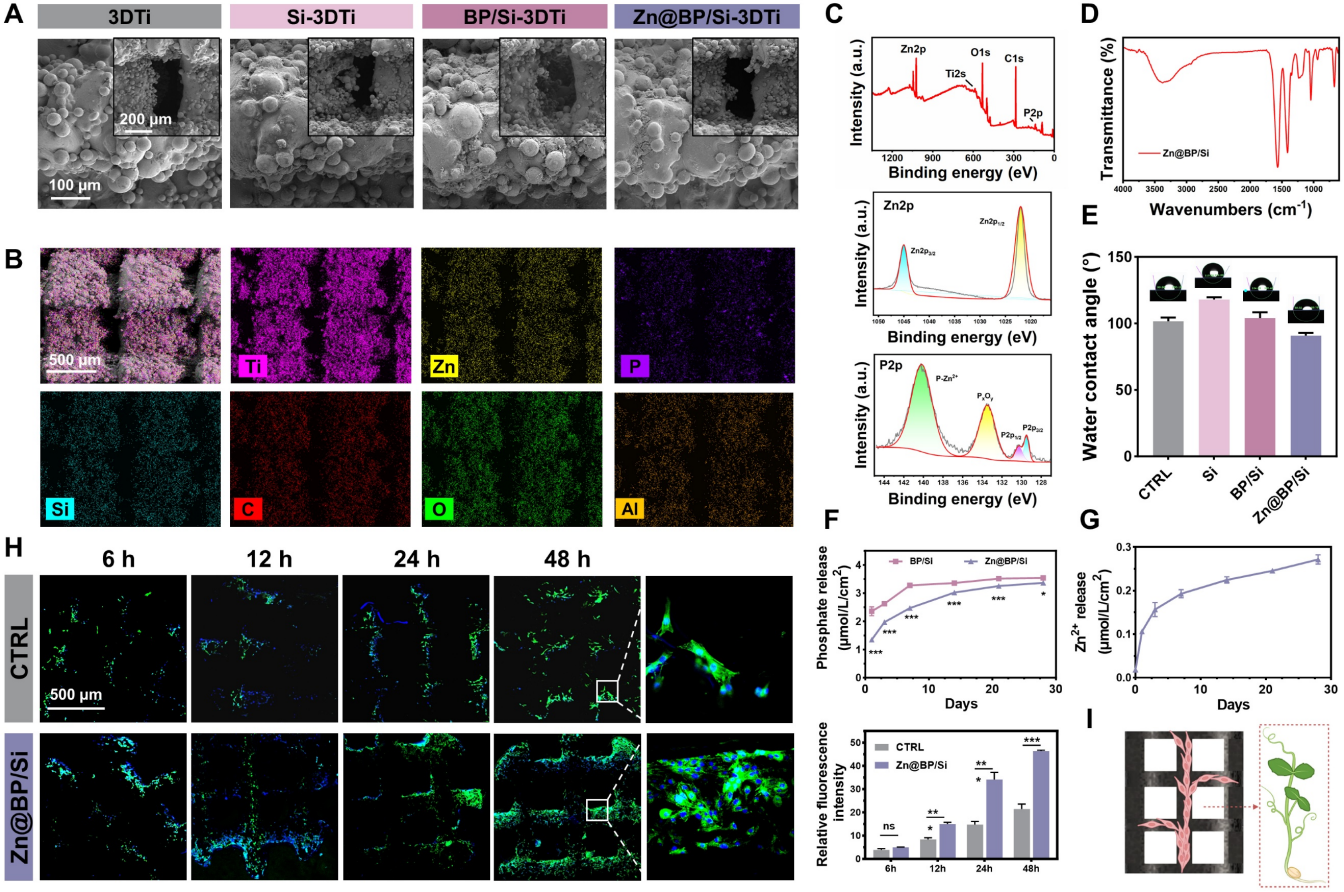
Construction and characterization of silanized Zn@BP-coated 3D printed Ti scaffolds. (A) SEM and (B) EDS mapping of blank, Si-coated, BP/Si-coated, and Zn@BP/Si-coated 3D Ti scaffolds. (C) XPS and high resolution spectra of Ti 2p and P 2p of Zn@BP/Si-coated 3D Ti scaffold. (D) FTIR spectra and (E) water contact angle of different coatings of 3D Ti alloy scaffolds. (F) Phosphate and (G) Zn^2+^ released from Zn@BP/Si-coated 3D Ti scaffolds after immersion in simulated body fluid solution (pH=5.4). (H) Confocal laser scanning microscope of HUVEC cell adhesion on the surface of Zn@BP/Si-coated scaffolds. (I) The schematic diagram of HUVEC adhering and growing to the scaffold like a vine. *P < 0.05, **P < 0.01, ***P < 0.001 compared with the CTRL group.

The healing of large segmental bone defects and fractures with significant misalignment or soft tissue embedding requires surgical intervention and the use of implants. Titanium and its alloys are preferred medical implant materials. 3D printing of precisely controlled porous structures can reduce the elastic mismatch between implants and bone, ensuring that both the macroscopic shape and microscopic structure meet the requirements of bone repair. We further modified porous scaffolds manufactured by selective laser melting technology by adding Zn^2+^-modified BP sheets. Porosity was approximately 70% (**Figure S1**). Static compression tests (**Figure S2**) disclosed an elastic modulus of 3.61 GPa, which is more compatible than titanium block with the elastic modulus of human cancellous bone (0.02-2 GPa). Scanning electron microscopy showed uniformly distributed laser-melted Ti-6Al-4V particles on the scaffold surface (**Figure 2A**). A uniformly thin film formed on the particle surfaces of the coated groups, and EDS mapping disclosed P, Zn, and Si signals distributed on the Zn@BP/Si surface (**Figure 2B**). XPS and FTIR confirmed that the -Si-O-Si- network formed by hydroxylated APTES on the alloy surface connected Zn@BP nanosheets to the substrate (**Figure 2C, D**). XRD showed diffraction peaks corresponding to the BP phase at (020), (040), and (060); Zn^2+^ at (103); and SiO_2_ at (002), (101), and (102) (**Figure S3**). Atomic force microscopy showed that the coating increased surface roughness (**Figure S4**), and that Zn@BP/Si exhibited the lowest contact angle among all groups, indicating the strongest hydrophilicity (**Figure 2E**). Peeling tests confirmed good adhesion of both silanized coatings (**Figure S5**).

We further evaluated the sustained release of phosphate and zinc ions, as well as the degradation of the coating (**Figure 2F, G**). Zn@BP/Si exhibited a slower and more stable release of phosphate than BP, and also yielded a sustained release of zinc ions over 28 days. Due to the acidity of the diabetic microenvironment, the sustained release of Zn@BP/Si was further tested under different pH conditions to observe the coating's degradation. Lower pH levels accelerated BP degradation and Zn^2+^ release (**Figures S6-8**).

The interaction between the Zn@BP/Si coating and cells was investigated. A CCK-8 assay disclosed that the Zn@BP/Si scaffold was highly biocompatible with HUVECs and BMSCs (**Figure S9**). After culturing on the scaffold, analysis of HUVEC cytoskeletons indicated that the number of cell adhesions on the Zn@BP/Si scaffold was significantly higher than that of the control group. The presence of perinuclear elongated actin filaments featuring a flattened spindle shape indicated that the coating enhanced cell adhesion and spreading in the early stage (**Figure 2H**). The surface of the Zn@BP/Si facilitates the adhesion and growth of HUVEC, just like the climbing of vines (**Figure 2I**).

### Antimicrobial properties of Zn@BP/Si coated 3D Ti alloy scaffolds

**Figure 3:**
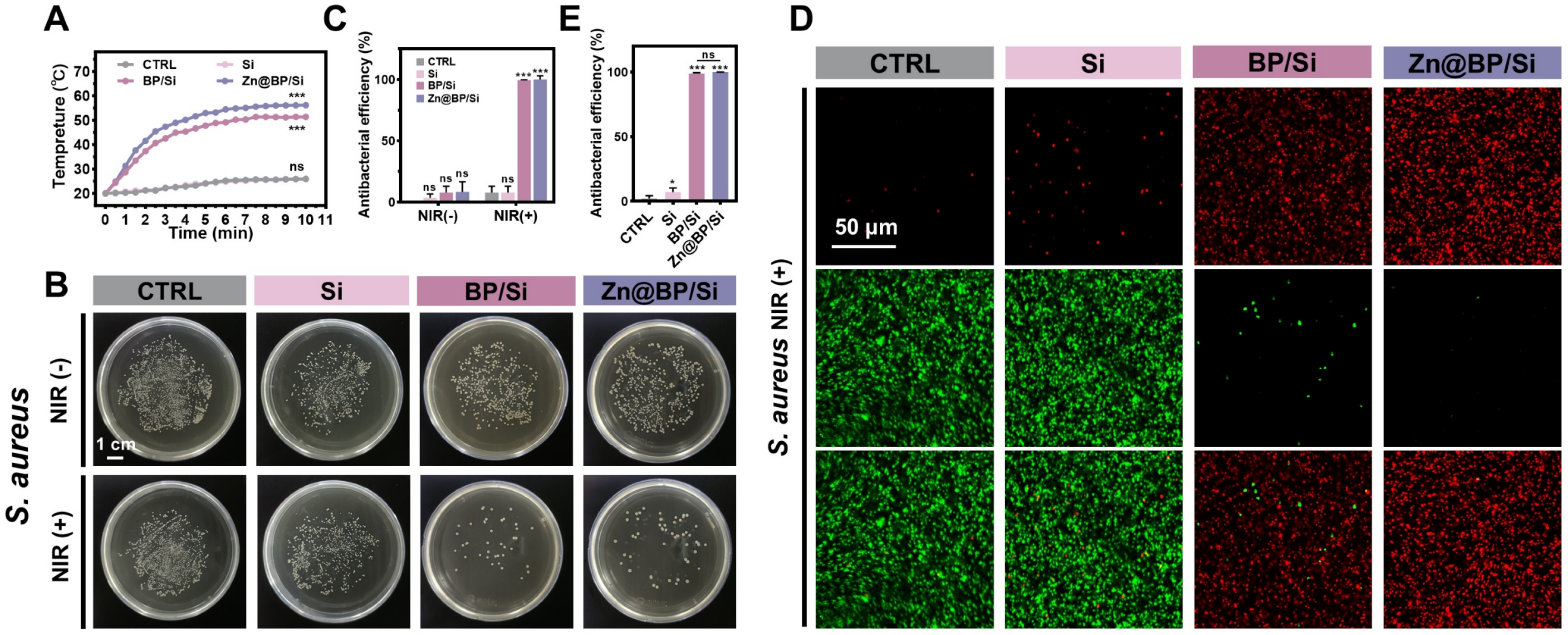
Antimicrobial activities of Zn@BP/Si-coated Ti alloy scaffolds. (A) Temperature curves of Zn@BP/Si-coated Ti alloy scaffolds with 808 nm NIR laser. (B, C) Culture and corresponding antimicrobial rates of *S. aureus* without or with NIR. (D, E) Live-dead bacterial staining of *S. aureus* after NIR exposure.

Hyperglycemia predisposes to infection, while implants with antibacterial properties enhance the healing of potentially infected bone lesions. **Figure 3A** and **Figure S10** show the photothermal performance and stability of different coatings under 1 W/cm² NIR. Temperature increase was higher for Zn@BP/Si than unmodified BP/Si (**Figure 3A**). Plate experiments showed that without NIR, both BP/Si and Zn@BP/Si inhibited the growth of *Escherichia coli*, while the other groups showed no antibacterial effect (**Figure S11**). Under NIR irradiation, both BP/Si and Zn@BP/Si were highly active against *Staphylococcus aureus* and *E. coli* (**Figure 3B, C, S11**). The live/dead bacterial fluorescence staining results were consistent with the plate experiments (**Figure 3D, E, S12**), confirming that Zn@BP/Si possesses excellent photothermal antibacterial properties *in vitro*.

### Zn@BP/Si promoted osteogenesis-angiogenesis coupling by mitigating HUVEC apoptosis

**Figure 4:**
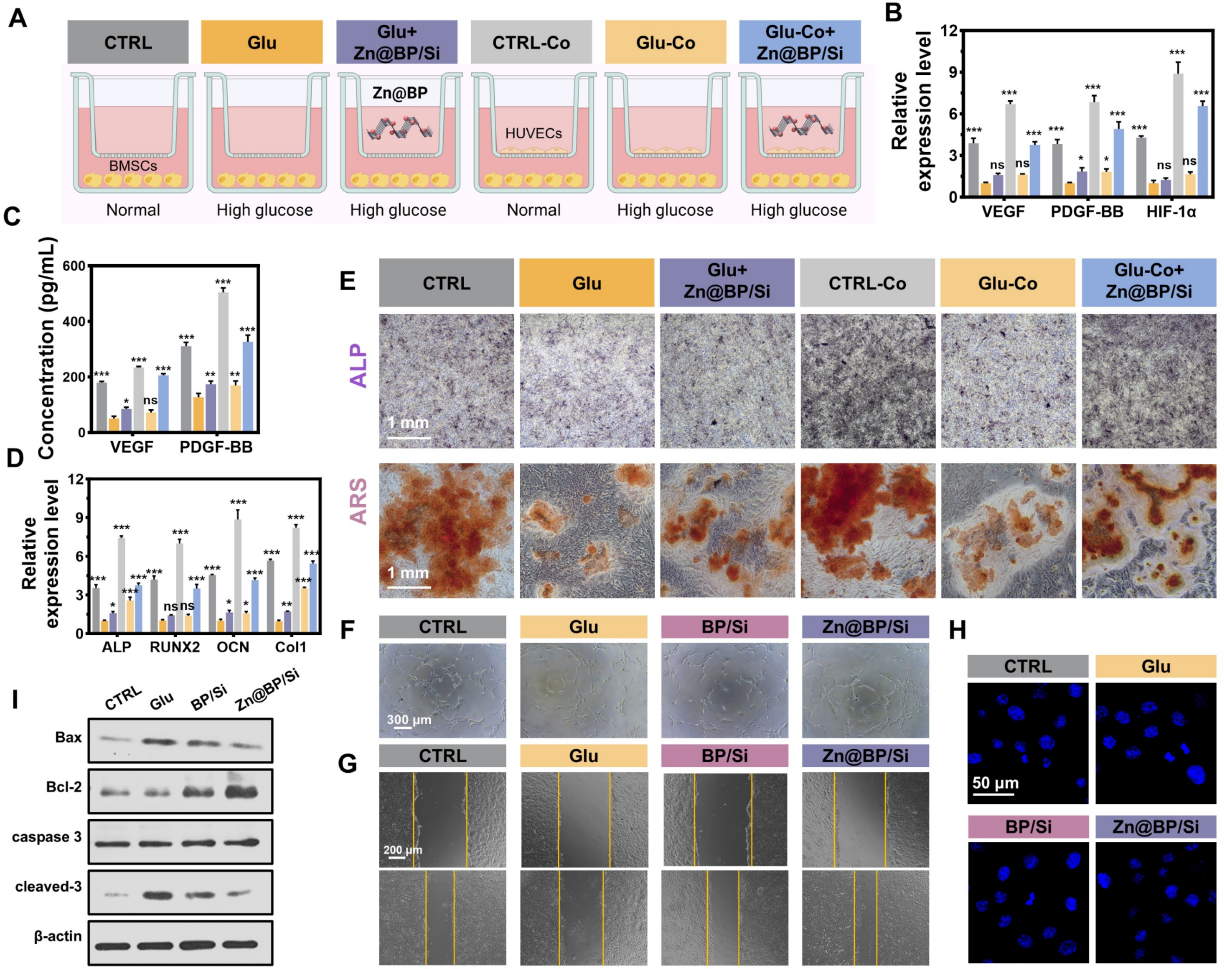
Zn@BP/Si promoted osteogenesis-angiogenesis coupling by mitigating HUVEC apoptosis *in vitro*. (A) Experimental design schematic diagram showing the co-culture of HUVECs and BMSCs in a transwell system. The concentration of the key angiogenesis-osteogenesis coupling factors determined by (B) qPCR and (C) ELISA in 24h and 48h. (D) mRNA expression levels of the key osteogenesis-related marker genes in BMSCs determined by qPCR in 3 days. Data are present mean ± SD (n = 3). The mean was represented by columns and the SD were represented by error bars. *P < 0.05, **P < 0.01, ***P < 0.001. (E) Images of BMSCs osteogenic differentiation on day 14 by ALP staining and the images and relative quantitative analysis of BMSC osteogenic differentiation on day 21 by ARS staining. (F) HUVEC tube formation assay after 6 h of culture in endothelial medium containing different coating extracts. (G) Scratch wound healing assay after 24 h of culture with extracts. (H) Hoechst 33258 staining was conducted to observe apoptotic cells. (I) Western blotting and quantitative analysis of Bax, Bal-2, caspase 3 and cleaved-3 expressions.

Osteoblasts and osteoclasts collaborate during the bone repair process. Both osteoblasts and osteoclasts play significant roles in the diabetic microenvironment of bone defects 9. In this study, we primarily focused on the functions of BMSCs, while Zn@BP/Si coating may also influence osteoclast differentiation to some extent, and appropriate activation of osteoclasts can further enhance the bone repair process. Given the controlled degradation, antibacterial properties, and biocompatibility of Zn@BP/Si, we further investigated its role in osteogenesis-angiogenesis coupling. Endothelial cells in the bone marrow can mobilize to the injury site, synthesizing and releasing specific angiocrine factors (VEGF, PDGF-BB, HIF-1α, *etc.*) to enhance osteogenic differentiation of mesenchymal stem cells and promote bone reconstruction through osteogenesis-angiogenesis coupling. However, endothelial cell function, including the promotion of coupling, declines under hyperglycemia. The effect of Zn@BP/Si on osteogenesis-angiogenesis coupling was observed by an indirect co-culture of HUVECs/BMSCs (**Figure 4A**). After 48 h, RT-qPCR was used to detect the gene expression of angiocrine factors by HUVECs in the upper chamber (**Figure 4B**). ELISA was used to detect the expression of coupling regulatory factors in the bottom chamber (**Figure 4C**). Both the gene expression in the upper layer and protein expression of coupling factors in the lower layer were reduced in the Glu group compared to controls. In contrast, the addition of Zn@BP/Si increased the expressions of related genes and proteins compared to those of the Glu group, indicating that Zn@BP/Si stimulates HUVECs to secrete osteogenesis-angiogenesis coupling factors. In terms of osteogenic gene expression, RT-qPCR on day 3 showed that BMSC *Runx2* and *ALP* expressions decreased in the Glu group compared to the control group, regardless of co-culture. In contrast, these pro-osteogenic genes were significantly upregulated in the Glu-Co+Zn@BP/Si group compared to the Glu-Co group (**Figure 4D**). ALP and ARS staining results of BMSCs in the lower chamber were consistent with osteogenic gene expression. The Zn@BP/Si group showed significantly higher ALP activity and larger mineralized nodules (**Figure 4E, S13**). Notably, we observed a slight but statistically insignificant increase of osteogenic differentiation in the Glu+Zn@BP/Si group compared to the Glu group. This indicates that the effect of Zn@BP/Si on the osteogenic differentiation of BMSCs was not entirely direct. Previous research reported that silicon ions has been shown to induce angiogenesis by upregulating NOS, leading to increased VEGF production, and play a critical role in bone mineralization 10, 11. Therefore, the Zn@BP/Si coating may combine the effects of Si and Zn@BP to enhances HUVEC-BMSC interactions by upregulating osteogenesis-angiogenesis coupling regulatory factors, and thereby may reverse hyperglycemia-induced osteogenic inhibition.

Oxidative stress promotes apoptosis and impairs differentiation and angiogenic function of endothelial cells, and thereby delaying bone healing in the setting of hyperglycemia. Increasing the survival rate of endothelial cells and prolonging their angiogenic function can enhance angiogenesis and promote osteogenesis - angiogenesis coupling. Cell tube formation was severely impaired in the Glu group compared to the control group. However, the numbers of branching points and tube lengths were significantly increased in the Zn@BP/Si group compared to the Glu group, demonstrating superior tube formation capability (**Figure 4F, S14**). Additionally, we found that, compared to the BP/Si group, the Zn@BP/Si group exhibited a significantly enhanced angiogenic effect, suggesting that Zn^2+^ modification can support and protect endothelial cell functions. Scratch assay results were consistent with the tube formation assay, indicating that Zn@BP/Si improved the angiogenic ability of HUVECs under high-glucose conditions (**Figure 4G, S15**). Hoechst-staining disclosed that HUVEC nuclear fragmentation was higher in the Glu group than in the control group (**Figure 4H**), but was mitigated by the addition of Zn@BP/Si. Western blotting disclosed that expressions of apoptosis-related proteins Bax and cleaved caspase-3 increased, while Bcl-2 decreased in the Glu group (**Figure 4I, S16**). In the Zn@BP/Si group, Bax and cleaved caspase-3 levels were lower, while Bcl-2 levels were higher, than in the Glu group, suggesting that Zn@BP/Si may inhibit apoptosis and restore angiogenic function, with Zn playing a key role in this process.

### Zn@BP/Si activated YME1L to mitigate mitochondrial fission and regulate mitochondrial dynamics

**Figure 5:**
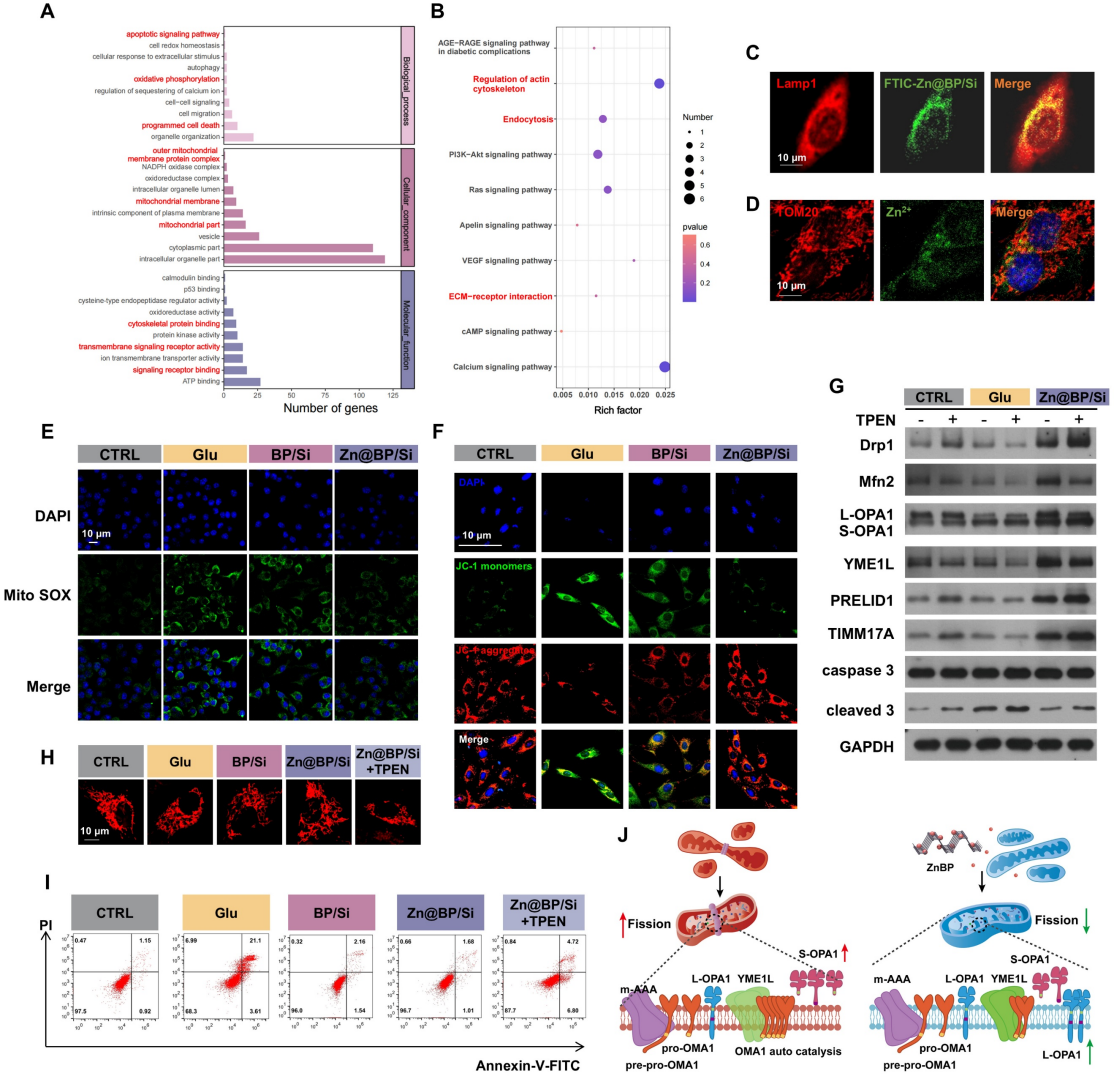
Zn@BP/Si activated YME1L to reduce abnormal mitochondrial fission to regulate mitochondrial dynamics. (A-B) GO and KEGG enrichment analyses revealed enrichment for biological processes. (C) Representative fluorescence images of FITC-labeled Zn@BP/Si endocytosis after Zn@BP/Si treatment for 10 min. Lysosomes were labeled with Lamp1. (D) Destination of Zn^2+^ in mitochondria after Zn@BP/Si exposures. (E) Mito SOX detected mtROS content and (F) JC-1 detected mitochondrial membrane potential of HUVECs to reveal mitochondrial function. (G) Western blotting detected expressions of YME1L, proteins related to mitochondrial fusion and division, and apoptosis. (H) Mito-tracker staining showed the morphological changes of mitochondria after zinc chelator TPEN was added. (I) Cells stained with the Annexin-V/PI dye and the apoptosis rates were analyzed by flow cytometry. *P < 0.05; **P < 0.01; ***P < 0.001. (J) The schematic diagram of regulation mitochondrial dynamics by upregulating YME1L activity via Zn@BP/Si.

mRNA sequencing analysis of HUVECs under the effect of Zn@BP/Si disclosed 514 differential genes between the Glu and Zn@BP/Si groups. GO and KEGG enrichment analyses revealed that gene expressions related to apoptosis and programmed cell death differed significantly (**Figure 5A-B**). Differential signaling pathways were mainly distributed in the regulation of cell structures such as the cell membrane and mitochondria. Therefore, we hypothesized that Zn@BP/Si may reduce HUVEC apoptosis by affecting mitochondria, and investigated its potential mechanism of action. We found that FTIC-labeled Zn@BP/Si was taken up by HUVECs and localized in lysosomes (**Figure 5C**). FluoZin-3 AM staining revealed that Zn^2+^ was localized on mitochondrial membranes 1 h after endocytosis, suggesting that Zn@BP/Si released Zn^2+^ during intralysosomal degradation, enabling consequent Zn^2+^ interactions with mitochondria (**Figure 5D**). Mitochondria are crucial participants in programmed cell death; their respiratory dysfunction, excessive fission and fusion, and abnormal autophagy processes can all cause apoptosis. Hyperglycemia promotes mitochondrial dynamics toward fission, resulting in fragmented and dysfunctional mitochondria. Consequently, the effects of Zn@BP/Si on ΔψM and mtROS levels were examined. Mito-SOX also detected an increase in mtROS levels under high glucose, indicating mitochondrial over-division (**Figure 5E**). JC-1 staining indicated a decrease in the red/green ratio in the Glu group, reflecting the significant reductions of mitochondrial membrane potential and function (**Figure 5F**). In contrast, in the Zn@BP/Si group, intracellular mtROS levels and mitochondrial membrane potential were recovered, suggesting that Zn@BP/Si can rescue dysfunctional mitochondria in endothelial cells. These observed differences between the Zn@BP/Si and BP/Si groups suggest that Zn modification of BP may underlie, at least in large part, the rescue of dysfunctional mitochondria in endothelial cells.

The upstream mechanism by which Zn@BP/Si rescued the dysfunctional HUVEC mitochondria was further investigated. The mitochondrial protease YME1L, a zinc metalloproteinase localized to the mitochondrial inner membrane, is involved in the regulation of mitochondrial protein quality. Zinc ions play a critical role in the regulation of its enzymatic activity. OPA, a member of the mitochondrial plastocyanin family, can be cleaved into long (L-OPA1) and short (S-OPA1) forms 12, 13. YME1L and OMA1 co-regulate OPA1 cleavage to balance the mitochondrial fusion and fission. YME1L prevents the development of abnormal mitochondrial cristae morphology and mitochondrial network fragmentation by regulating the cleavage of L-OPA1. Western blotting showed increased expression of the mitochondrial fission-related protein Drp1 and S-OPA1 accumulation, and reductions of the fusion-related proteins Mfn1 and Mfn2 in the Glu group. Although the expression of the upstream regulator YME1L was basically the same, the abundance of its substrates PRELID1 and TIMM17A increased (**Figure 5G, S17**). Conversely, in the Zn@BP/Si group, the substrates of YME1L decreased, while the product L-OPA1 and mitochondrial fusion proteins increased, and fission-related proteins decreased. This indicates that the catalytic activity of YME1L was reduced under high glucose, leading to OPA1 dysregulation, the decreased expression of mitochondrial fusion proteins, and the imbalance in mitochondrial fusion-fission. Zn@BP/Si enhanced the catalytic activity of YME1L, prevented excessive turnover of OPA1, and improved mitochondrial dynamics to mitigate mitochondrial dysfunction under high glucose conditions.

After the addition of the zinc chelator TPEN, the expression of the substrate in the Zn@BP/Si group increased again. Meanwhile, L-OPA1 expression decreased, while S-OPA1, DRP1, and apoptosis-related proteins increased. Immunofluorescence showed that relatively intact mitochondria fragmented (**Figure 5H, S18**). Flow cytometry also revealed that TPEN antagonized the Zn@BP/Si-mediated reduction of apoptosis, and cell survival decreased (**Figure 5I**).

This confirmed that Zn@BP/Si may regulate mitochondrial dynamics by upregulating YME1L activity, further mitigating mitochondrial dysfunction and consequent HUVEC apoptosis and dysfunction. High glucose conditions impair dietary zinc absorption and tissue uptake. The Zn@BP/Si coating, after degradation and endothelial cellular uptake, slowly degraded to Zn^2+^, which may recover YME1L activity, inhibit abnormal mitochondrial fission, and rescue apoptosis. Our results are consistent with the concept that endothelial cell function is highly sensitive to mitochondrial homeostasis 14. The restoration of mitochondrial homeostasis is crucial for the regeneration of blood vessels and bone in areas of osseous defects. The results demonstrate that Zn@BP/Si promotes the growth of “vascular vines” by inhibiting excessive mitochondrial fission.

### Zn@BP/Si promoted osteogenesis-angiogenesis coupling to accelerate femoral bone regeneration

**Figure 6:**
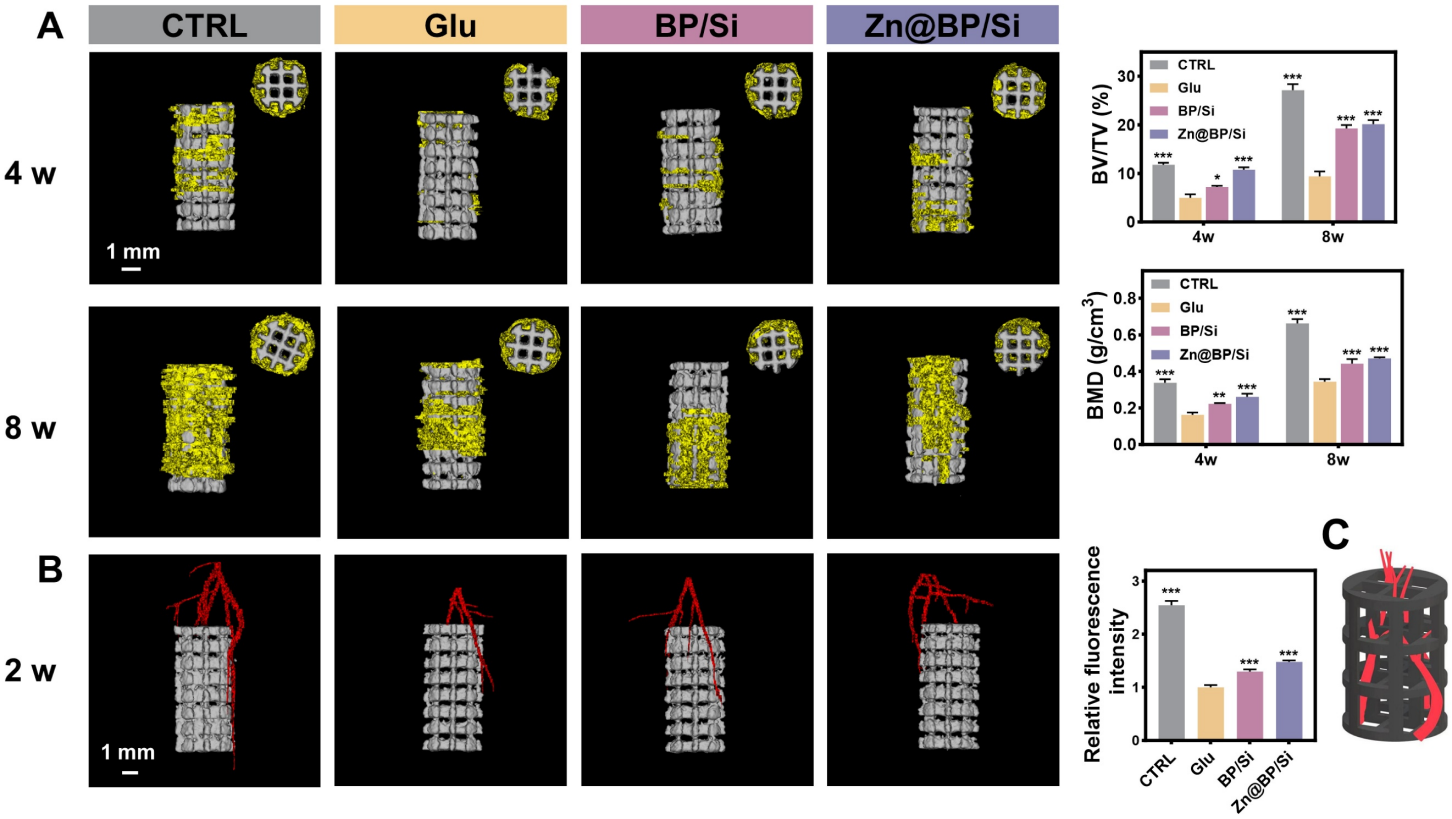
*In vivo* osteogenic effect of 3D-printed titanium alloy scaffolds with Zn@BP/Si coating. (A) Representative 3D micro-CT images at 4 and 8 weeks postoperation. Quantitative analysis of micro-CT reconstructions of different scaffolds at 4 and 8 weeks postoperation (n = 3 in each group). (B) 3D image reconstruction of vascular distribution and the quantitative analysis of blood vessel quantification. *P < 0.05, **P < 0.01, ***P < 0.001. (C) The diagram of Zn@BP/Si facilitating the infiltration of blood vessels into the scaffold like vines.

Due to the significant efficiency of the Zn@BP/Si coating in inducing vascularized bone regeneration and its excellent photothermal antibacterial activity, we further explore its therapeutic effects on bone regeneration *in vivo*. We injected *S. aureus* into diabetic rats to simulate the potential risk of infection. All animals were observed for their behavior and body weight to confirm that there were no significant adverse effects on the rats. Infrared thermal imaging showed that after 5 minutes of NIF irradiation, the temperature of Zn@BP/Si group at the local defect site rose to 44.7 ºC (**Figure S19**). Micro-CT reconstructions of the implants and bone tissue were performed at 4 and 8 weeks post-surgery (**Figure 6A**). New bone tissue (yellow) can be observed growing into the scaffold (white). In diabetic group, less new bone formation was detected in the peripheral area outside implant, with BV/TV significantly lower than that in the control group. The Zn@BP/Si group showed a higher level of new bone formation at both 4 and 8 weeks. At 8 weeks, more new bone tissue approached the longitudinal axis of the implant in the Zn@BP/Si and CTRL group, while the other groups remained limited to the peripheral area of the implant. At 2 weeks, the vessels in the Zn@BP/Si and CTRL group were denser and thicker compared with the sparse ones in the uncoated and Si-coated groups. (**Figure 6B**). Blood vessels grow into the scaffold like vines, forming a vascular network to construct “branches” that promote the recruitment and differentiation of osteoblasts as “leaves” (**Figure 6C**). These results indicated that Zn@BP/Si-coated Ti alloy promoted vascularized bone regeneration in diabetic femoral bone defect. Furthermore, live and dead bacteria staining results showed that the number of bacteria in the Zn@BP/Si group was significantly lower than in the Glu group, suggesting that Zn@BP/Si not only promoted diabetic femoral bone regeneration but also prevented *S. aureus* infection (**Figure S20**).

**Figure 7:**
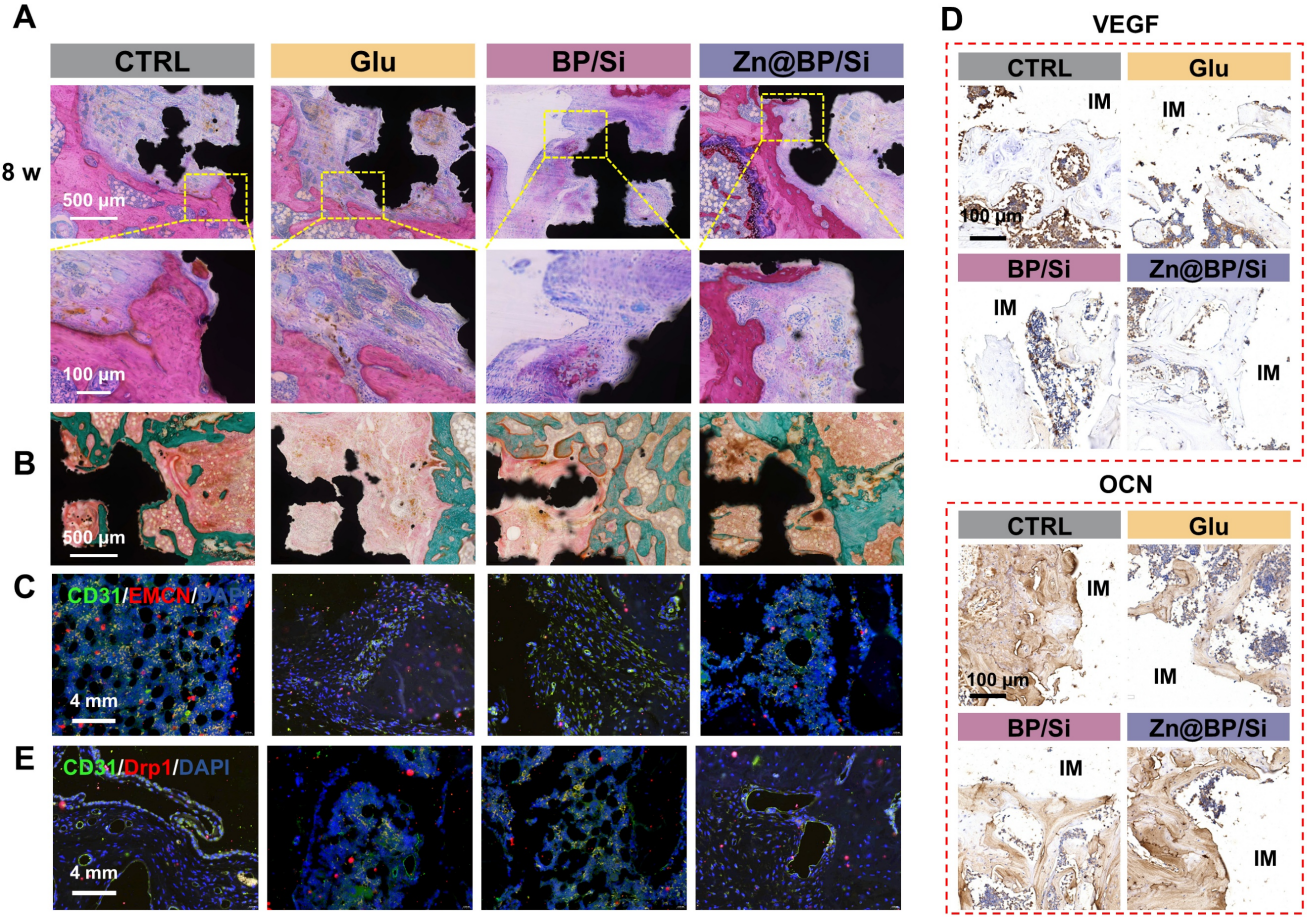
Zn@BP/Si promoted vascularized bone regeneration by inhibiting HUVEC apoptosis by mitochondrial dynamics regulation. (A) Represent images of H&E staining. (B) Represent image of Masson staining. Green represents new bone (collagen fibers and cartilage) and red represents muscle fibers, fibrin and red blood cells. (C) CLSM images of the immune-stained tibia sections displayed type H vessels in the diabetic femoral bone defect and histomorphometric quantification of the type H vessel columns. CD31 (green) and EMCN (red). (D) Immunohistochemistry staining of VEGF and OCN proteins around the implants at 8 weeks (red arrows indicate the osteoclasts). (E) Immunofluorescent staining of CD31 and Drp1.

H&E and Masson trichrome staining images were shown in **Figures 7A-B**. A small amount of bone tissue and a large amount of fibrous tissue generated in the Glu group, whereas the Zn@BP/Si group exhibited more newly formed bone tissue with favourable implant osseointegration. Masson staining also revealed that the area around the scaffold was filled with green-red new bone and collagen in the Zn@BP/Si group, with scattered relatively mature calcification points. At 2 weeks, immunofluorescence staining showed that the proportion of type H vessels in the Zn@BP/Si group was significant increased (**Figure 7C**). Immunohistochemical staining demonstrated a significant upregulation of OCN and OPN proteins in the Zn@BP/Si group (**Figure 7D**). These results suggested that Zn@BP/Si promoted early angiogenesis and osteogenesis-angiogenesis coupling *in vivo*. Further immunofluorescence results in **Figure 7E** revealed that the expression of Drp1 in CD31-positive cells was significantly elevated in the Glu group but reduced in the Zn@BP/Si group. This confirmed that Zn@BP/Si rescued high glucose-induced apoptosis and functional decline in HUVECs and promoted the osteogenesis-angiogenesis coupling in diabetes, by regulating mitochondrial dynamics and preventing excessive mitochondrial fission.

## Conclusion

Drawing inspiration from the growth pattern of vines, the Zn@BP/Si coating on the 3D-printed titanium scaffold was developed to promote the coupling of angiogenesis and osteogenesis. This modification improved the stability and photothermal performance of BP, conferred antibacterial activity, and accelerated the infiltration of vascular and bone tissues within the scaffold. The mechanism of action comprised Zn@BP/Si-mediated enhancement of HUVEC-BMSC interactions and upregulation of osteogenesis-angiogenesis coupling regulators to reverse high glucose-induced osteogenic inhibition. Zn@BP/Si activated the mitochondrial protease YME1L, reduced abnormal mitochondrial fission and provided motivation for cell adhesion and migration, just like the climbing of vines.

The results demonstrate that the Zn@BP/Si promotes the growth of “vascular vines” and ameliorates the angiogenic and osteogenic inhibition in diabet. In summary, we found that the bio-inspired Zn@BP/Si coating offers a potential therapy of diabetic bone defects by regulating mitochondrial dynamics, thus providing a new strategy for the modification of metallic implants.

## Experimental section

### Preparation and characterization of BP and Zn@BP

Based on the previous study, bulk BP was exfoliated using liquid-phase separation in N-methylpyrrolidone (NMP) to synthesize BP nanosheets 15. The obtained BP was prepared as a 0.5 mg/mL aqueous suspension. 11.62 mg of zinc acetate powder was added to 5 mL BP suspension and mixed, followed by sonication in an ice bath for 3 minutes, and then stirred at 350 rpm for 3 hours to collect Zn@BP by centrifugation. The Zn@BP was washed with anhydrous ethanol and deionized water, and the precipitate was collected by centrifugation and stored in a dark N_2_ atmosphere for subsequent experiments. Scanning electron microscopy (SEM, Zeiss Sigma 300, Germany) and high-resolution transmission electron microscopy (HRTEM, Tecnai G2 F20 S-TWIN, USA) were used to observe the morphology of nanomaterials. Fourier Transform Infrared Spectrometer (FTIR, TENSOR27, German) and raman spectroscopy (Renishaw, UK) with a wavelength of 513 nm was employed to characterize the functional groups and structure. X-ray photoelectron spectroscopy (XPS; Thermo Fisher Scientific, UK) was used to identify the elemental composition and chemical bonds of Zn@BP, with the C1s peak binding energy calibrated to 284.6 eV. The zeta potential was measured using a Zeta potential analyzer (Litesizer 500). The prepare of FITC-Zn@BP was based on the previous method 16, and APTES was used to functionalize the surface of Zn@BP to attach FITC molecules.

### First principles calculation methods

All first-principles DFT calculations were performed with the Vienna ab initio simulation package (VASP) 17 using the Perdew-Burke-Ernzerhof generalized gradient approximation (PBE-GGA) 18 method. All calculations include spin-polarization. A monolayer black phosphorus surface was prepared for a 3×3×1 unit cell with a 15 Å vacuum layer along the normal z direction. The plane-wave cutoff energy was set to 400 eV. A 2×3×1 Monkhorst-Pack k-point grid is employed in the calculations. The adsorption energy (E_ads_) was calculated as follows: E_ads_ = E_Zn-BP_ - E_Zn_ - E_BP_, where E_Zn-BP_ is the energy of Zn atom adsorbed on P surface, E_Zn_ is the energy of Zn^2+^ and E_BP_ is the energy of P surface. VASPsol package was used with the default dielectric constant of ε = 78.4 to include implicit solvation 19.

### Preparation and characterization of 3D printed Ti alloys with coatings

Selective laser melting was used to manufacture Shape3D-designed porous titanium scaffolds with a width of 300 μm, average pore size of 500 μm, and 65.3% porosity. Preheated spherical Ti6Al4V metal powder, with a particle size range of 15-53 μm, was used to produce cylindrical porous samples (Ø3 mm × 6 mm) by high-energy laser melting under vacuum conditions according to layered data planning routes. The printer operated at a power of 280 W, with a powder layer thickness of 30 μm, a scan spacing of 0.1 mm, and a scan speed of 1000 mm/s. The printed samples were ultrasonically cleaned and dried at 50 ℃. The mechanical properties related to the implantation of solid titanium alloy rods and 3D-printed scaffolds were evaluated using an electronic universal compression testing machine. The loading speed was set at 0.5 mm/min, and the loading duration was set at 200 seconds (or until the stress-strain curve no longer changed).

The printed scaffolds were soaked in 1 mol/L sodium hydroxide at 60 ºC for 20 minutes and then rinsed with deionized water. BP or Zn@BP was added to 1 mL of 5% hydrolyzed γ-Aminopropyl triethoxysilane (APTES) (APTES/anhydrous ethanol = 1:19) to achieve a final concentration of 0.5 mg/mL in the solution. The samples were then immersed in the solution after adding a crosslinking agent [95.7 mg 1-(3-Dimethylaminopropyl)-3-ethylcarbodiimide (EDC) and 286.25 mg N-Hydroxy succinimide (NHS)]. This mixture was continuously stirred (400 rpm) at room temperature for 24 hours to obtain silanized coatings, referred to as the Si group, BP/Si group, and Zn@BP/Si group, respectively. The composition and structure of the different coatings are characterized.

### Degradation and biocompatibility test

The samples were soaked in simulated body fluid (pH = 7.4 or pH=5.4) at 37 ºC for 1, 3, 7, 14, 21, and 28 days, with the soaking solution being replaced daily. Inductively coupled plasma optical emission spectrometry (ICP-OES, Agilent 720, USA) was used to measure the concentrations of Zn, P, and Si after degradation.

BMSCs and HUVECs were cultured in Dulbecco's Modified Eagle's Medium (DMEM) or ECM containing 10% fetal bovine serum and 1% penicillin-streptomycin solution. The culture environment was maintained at 5% carbon dioxide and a constant temperature of 37 ºC. To construct a diabetes-inducing medium, dextrose (Sigma, 25 μM) and palmitate (Sigma, 500 μM) were added and labeled as “Glu.” The extracts of the different coating were obtained according to the previous studies 20, 21. Cells (1×10^4^) were co-cultured with different extracts for 3 days, and cell proliferation was evaluated using a CCK-8 kit at the wavelength of 450 nm. HUVECs were seeded into 12-well plates containing different scaffolds and cultured for 6, 12, 24, and 48 hours. After fixation and permeabilization, FITC-phalloidin (100 nM) and DAPI (10 µg/mL) were used for staining F-actin and nuclei to evaluate the effect of different coated scaffolds on cell adhesion.

### Photothermal performance texting *in vitro*

An 808 nm near-infrared laser (1 W/cm²) irradiated the coated surface for 10 minutes, recording temperature changes using an infrared thermal imager. The laser was activated/deactivated every 5 minutes. *Staphylococcus aureus* and *Escherichia coli* were cultured in fresh Luria-Bertani liquid medium at 37 ºC. Bacteria in the logarithmic growth phase were mixed with the extracts from each group in 200 μL of saline, and the suspensions were transferred to 24-well plates and incubated for 48 hours. Each group was subjected to near-infrared laser irradiation for 10 minutes and then sonicated in phosphate buffered saline for 5 minutes. The resulting bacterial suspensions were diluted and cultured to quantify colony-forming units (CFU). Live/dead bacterial staining was performed using the MycoLight™ rapid fluorescence gram-positive bacteria staining kit (AAT Bioquest Inc., USA), and the fluorescence of bacteria was monitored using a fluorescence microscope.

### Transwell

Using 24-Transwell plates with a pore size of 0.4 μm (Corning Life Sciences), BMSCs seeded in the bottom chamber and HUVECs in the top. The CTRL group was set with only BMSCs seeded in the bottom chamber and an empty top chamber. The Ctrl and Ctrl-Co groups use normal medium, while others use Glu medium. At 24 hours, RT-qPCR was used to detect the gene expression of angiogenesis-osteogenesis coupling factors of HUVECs in the upper chamber, and ELISA was used to detect coupling regulatory factors in the culture medium of the bottom chamber at 48 hours. mRNA expression levels of the key osteogenesis-related marker genes in BMSCs determined by qPCR in 3 days. To compare the effects of osteogenic differentiation and mineralization, an alkaline phosphatase staining kit (Beyotime Biotechnology, China) was used on day 14, and an Alizarin Red S staining kit (Cyagen Biotechnology Co., Ltd., China) was used on day 21 to stain the BMSCs in the bottom chamber. The cells were fixed with 4% paraformaldehyde and stained according to the manufacturer's instructions. Images were captured and quantified using Image J.

### *In vitro* angiogenesis evaluation

For the tube formation assay, 1 mL of extract was added to the HUVECs overnight. Matrigel (BD Biosciences, USA) was added to a 96-well plate and incubated for 30 minutes. Then, HUVECs at a concentration of 2×10^5^ cells/mL were seeded onto the Matrigel-coated wells. After 6 hours, the main segment length and the number of nodes and mesh structures were analyzed using an optical microscope (Observer7, Zeiss, Germany) and Image J. HUVECs seeded in 12-well plates were subjected to the scratch wound healing assay using the corresponding extracts. When the cells reached 70% confluence, a sterile pipette tip (1 mL) was used to scratch the bottom surface of the wells. Images were collected at 0 and 24 hours using a stereomicroscope (Leica, Germany) to analyze wound closure.

### Reverse transcription-polymerase chain reaction (RT-qPCR)

Total RNA of cells in different groups was extracted using Trizol, chloroform, and isopropanol. The RNA was then reverse-transcribed into cDNA according to the manufacturer's instructions. Quantitative PCR was performed using SYBR Premix Ex Taq (Takara Biotechnology, Japan). The relative mRNA expression levels of *VEGF, PDGF-BB, HIF-α, ALP, RUNX2, OCN,* and *Col1* were calculated and normalized to the expression level of glyceraldehyde-3-phosphate dehydrogenase (*GAPDH*). The primer sequences used are listed in Supplementary Appendix Table S1.

### RNA sequencing and untargeted metabolomics assay

The total RNA was extracted using a Trizol reagent (Invitrogen, CA, USA). The RNA purity and concentration were then assessed by means of a NanoDrop 2000 spectrophotometer (Thermo Scientific, USA). The RNA-seq library was then sequenced using the NovaSeq X plus sequencer. The transcriptome sequencing and analysis were performed by Majorbio Bio-pharm Technology Co., Ltd (Shanghai, China).

### Western blotting

Cell proteins were extracted using RIPA lysis buffer (Santa Cruz, USA) and quantified with a BCA protein assay kit (Thermo Scientific, USA). Different samples were separated by sodium dodecyl sulfate-polyacrylamide gel electrophoresis and transferred onto polyvinylidene fluoride membranes. After blocking with BSA, the membranes were incubated overnight at 4 ºC with primary antibodies [anti-Bax (Abcam, 1:5000); anti-Bcl-2 (Abcam, 1:2000); anti-caspase-3 (Abcam, 1:2000); anti-cleaved caspase-3 (Abcam, 1:5000); anti-YME1L (Proteintech, 1:1000); anti-Drp1 (Abcam, 1:1000); anti-Mfn2 (Proteintech, 1:20000); anti-OPA1 (Abcam, 1:2000); anti-PRELID1 (Abcam, 1:500); anti-TIMM17A (Proteintech, 1:20000); anti-β-actin (Proteintech, 1:500000)]. After washing with TBST, the membranes were incubated with horseradish peroxidase-conjugated anti-rabbit IgG or anti-mouse IgG (1:2000, Cell Signaling Technology, USA). Protein bands were detected and analyzed using the Tanon 5200 system (China) and enhanced chemiluminescence solution (Millipore, USA).

### Cell apoptosis assay

HUVEC in 6-well plates were treated with the Annexin V-FITC/PI Apoptosis Kit and analyzed by flow cytometry. HUVEC in 24 well plates (1×10^6^ cells per well) were stained with Hoechst 33258 (Beyotime) and visualized under a fluorescence microscope.

### Mitochondrial morphology, dynamics and functions

After 24 hours of culture with extracts, cells were incubated in serum-free DMEM containing 100 nM MitoTracker Red (Molecular Probes, USA). Mitochondrial morphology and networks were then observed using a laser confocal microscope, and the quantitative assessments were performed using the MiNA (Mitochondrial Network Analysis) toolset and MitoAnalyzer plugin in ImageJ. HUVECs were treated with FITC-Zn@BP extracts for 10 min, or Zn@BP extracts for 1 hour and then incubated with FluoZin-3 AM (3 nM) for 1 hour. The cells were fixed, permeabilized, and blocked. Then incubated overnight at 4 ºC with antibodies against Lamp1 or TOM20 (dilution 1:100), followed by incubation with Alexa Fluor 594 anti-rabbit antibody (dilution 1:100) for 1 hour. HUVECs were stained using the JC-1 staining detection kit (Servicebio, China) for 20 minutes. 2.5 μM MitoSOX Green (Molecular Probes) and 150 nM Mitotracker Red (Molecular Probes) were used to stain for 30 minutes. Images were captured using a laser confocal microscope, and the corresponding fluorescence intensity was quantified and measured using ImageJ software.

### *In vivo* bone augmentation evaluation

The Animal Experimental Ethics Committee of Guangzhou seyotin Biotechnology Co., LTD approved the animal procedures performed in this study (SYT2024037). After being fed a high-fat diet for 4 weeks, the rats were fasted overnight for 12 hours, followed by an injection of STZ at 35 mg/kg. Fasting blood glucose levels were measured at 3 days, 1 week, and 2 weeks after the STZ injection. A blood glucose concentration higher than 11 mmol/L on all three occasions indicated successful modeling of type 2 diabetes. While under anesthesia, a cylindrical hole (3 mm in diameter, 6 mm deep) was drilled into the distal femur of the diabetic rats to establish a femoral defect model. Implanted the uncoated, BP/Si-, or Zn@BP/Si- coated 3D-printed titanium alloy scaffolds into the defect and sutured the wound. After 2, 4, and 8 weeks, the rats were euthanized, and the implants, along with adjacent femoral bone, were removed and decalcified using ethylenediaminetetraacetic acid (EDTA). Fresh tissues were fixed with 4% PFA. Bone tissues were decalcified with 10% EDTA for 21 days and then embedded in paraffin. Longitudinal bone sections with the thickness of 4 μm were used for immunostaining. Assessments of angiogenesis and bone integration were conducted using Micro-CT scanning (Quantum GX2, Revvity, USA; 70 kV, 100 μA), 3D reconstruction, and histological analysis (including Hematoxylin and Eosin (H&E), Masson trichrome (methyl green), and immunofluorescence of CD31, EMCN, and Drp1, as well as OCN and OPN immunohistochemical staining. Analysis 14.0 was used to calculate bone volume per total volume (BV/TV) and bone mineral density (BMD).

### Statistical analyses

Data were expressed as mean ± standard deviation (SD) for n ≥ 3. Statistical analyses were performed using one-way ANOVA with post hoc Tukey's test or Student's t-test via GraphPad software. P-values < 0.05 were considered statistically significant.

## Supplementary Material

Supplementary experimental section, figures and table.

## Abbreviations

APTES: γ-Aminopropyl triethoxysilane
BMD: bone mineral density
BMSCs: bone marrow stem cells
BP: black phosphorus
BV/TV: bone volume per total volume
CFU: colony-forming units
DMEM: Dulbecco's Modified Eagle's Medium
*E. coli*: *Escherichia coli*
EDC: 1-(3-Dimethylaminopropyl)-3-ethylcarbodiimide
EDS: Energy dispersive X-ray spectroscopic
EDTA: ethylenediaminetetraacetic acid
FTIR: fourier transform infrared spectrometer
GAPDH: glyceraldehyde-3-phosphate dehydrogenase
HUVECs: human umbilical vein endothelial cells
ICP-OES: inductively coupled plasma optical emission spectrometry
NHS: N-Hydroxy succinimide
NIR: near-infrared
NMP: N-methylpyrrolidone
PBE-GGA: Perdew—Burke—Ernzerhof generalized gradient approximation
RT-qPCR: reverse transcription-polymerase chain reaction
*S. aureus*: *Staphylococcus aureus*
SD: standard deviation
STEM: scanning transmission electron microscopy
TEM: transmission electron microscopy
VASP: Vienna ab initio simulation package
XPS: X-ray photoelectron spectroscopy
XRD: X-ray diffraction
