# Supplementary material for: Vine-inspired zinc-ion modified black phosphorus coating accelerates bone tissue infiltration of 3D printed scaffolds
Source: Theranostics. 2025 Apr 9;15(11):5073–86. doi: 10.7150/thno.113623 (PMC12036863; doi:10.7150/thno.113623)
Supplement: Supplementary file 1 — Supplementary experimental section, figures and table. [file thnov15p5073s1.pdf]

Supporting Information

Vine-inspired zinc-ion modified black phosphorus coating accelerates bone tissue infiltration of 3D printed scaffolds

*Dan Li, Danni Dai, Jianrong Wang, Yan Wang, Yujia Tian, Chao Zhang**

Supplementary experimental section

Materials

Black phosphorus powder, zinc acetate powder, dextrose, palmitate, N-methylpyrrolidone (NMP), (3-Aminopropyl) triethoxysilane (APTES), sodium hydroxide, and anhydrous ethanol were purchased from Sigma-Aldrich (USA). Fluorescein 5-isothiocyanate (FITC) was obtained from MedChemExpress (Shanghai, China). MC3T3-E1 cells and human umbilical vein endothelial cells (HUVECs) were obtained from Cyagen Biotechnology Co., Ltd. (Guangzhou, China). MitoSOX Green, Dulbecco's modified Eagle's medium (DMEM), fetal bovine serum (FBS), and penicillin/streptomycin (100 units per ml) were obtained from Thermo Fisher Scientific (Cleveland, OH, USA). Endothelial Cell Medium (ECM) was purchased from ScienCell Research Laboratories, Inc. (USA). EDTA/trypsin (0.25%) was obtained from Gibco (Grand Island, NY, USA). *Staphylococcus aureus* and *Escherichia coli* were purchased from Shanghai Yingxin laboratory equipment Co., Ltd. Luria-Bertani medium was composed of tryptone 10 g/L, yeast extract 5 g/L, and sodium chloride 10g/L according to the formula. The cell counting kit-8 (CCK-8) was purchased from Dojindo Molecular Technologies (Inc., Kumamoto, Japan). Paraformaldehyde (4%; PFA) and 0.5% Triton X-100 were purchased from Biosharp Ltd (Hefei, China). The cell counting kit-8 (CCK-8) was obtained from Dojindo Molecular Technologies (Inc., Kumamoto, Japan). MitoTracker (red) and LysoTracker (green) were purchased from Beyotime Biotechnology (Shanghai, China). Simulated body fluid, phosphate buffered saline, 4′,6-diamidino-2-phenylindole (DAPI), and Calcein AM/PI Double Stain Kit were purchased from Solarbio Technology Ltd (Beijing, China).

Table S1 PCR primers for genes.

Gene	Primer sequence
VEGF-F	5'-AGGGCAGAATCATCACGAAGT-3'
VEGF-R	3'-AGGGTCTCGATTGGATGGCA-5'
PDGFB-F	5'-CTCGATCCGCTCCTTTGATGA-3'
PDGFB-R	3'-CGTTGGTGCGGTCTATGAG-5'
HIF-1α-F	5'-GAACGTCGAAAAGAAAAGTCTCG-3'
HIF-1α-R	3'-CCTTATCAAGATGCGAACTCACA-5'
ALP-F	5'-TCCGTGGGCATTGTGACTAC-3'
ALP-R	3'-TGGTGGCATCTCGTTATCCG-5'
RUNX2-F	5'-GACTGTGGTTACCGTCATGGC-3'
RUNX2-R	3'-ACTTGGTTTTTCATAACAGCGGA-5'
OCN-F	5'-GGTAGTGAACAGACTCCGGC-3'
OCN-R	3'-GGCGGTCTTCAAGCCATACT-5'
COL1A1-F	5'-CTGGCGGTTCAGGTCCAAT-3'
COL1A1-R	3'-TTCCAGGCAATCCACGAGC-5'

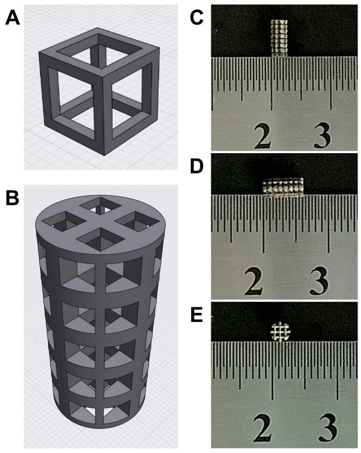

Figure S1. 3D titanium alloy scaffold prepared by SLM. (A) The single cell of the scaffold. (B) 3D modelling design drawing of the scaffold. (C-E) Pictures of different viewpoints of the scaffold.

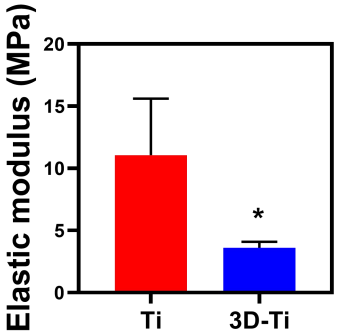

Figure S2. Modulus of elasticity of solid titanium rods and 3D printed titanium scaffolds calculated by static compression tests. *$P < 0.05$.

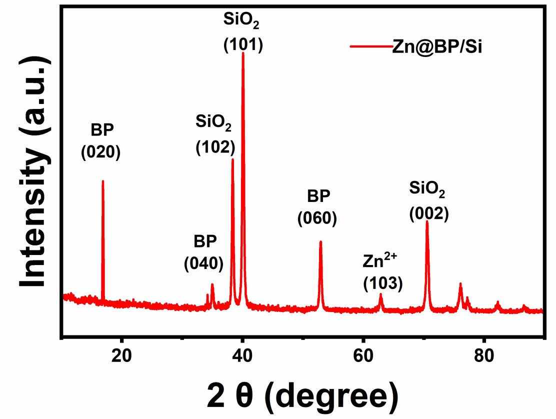

Figure S3. XRD patterns of Zn@BP/Si coated titanium alloys.

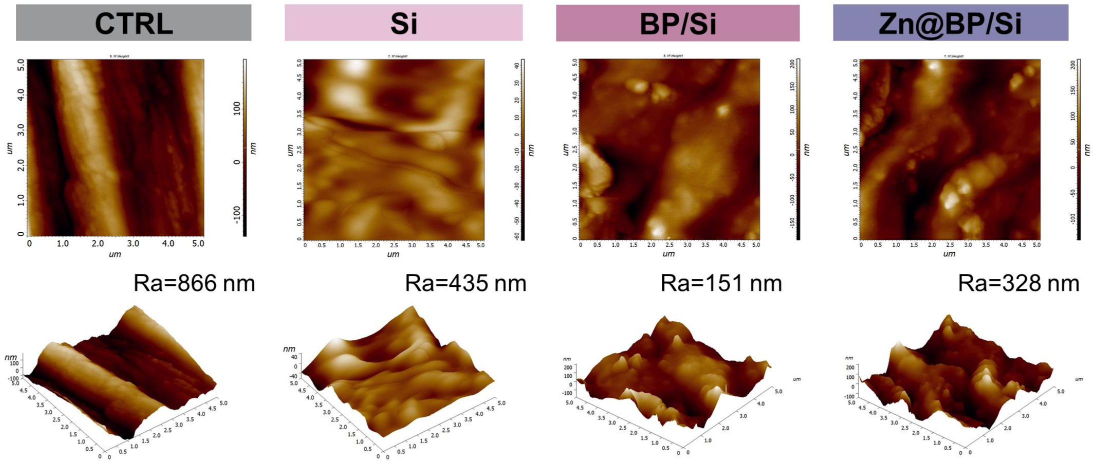

Figure S4. Surface morphology of titanium alloys with different coatings was examined by AFM.

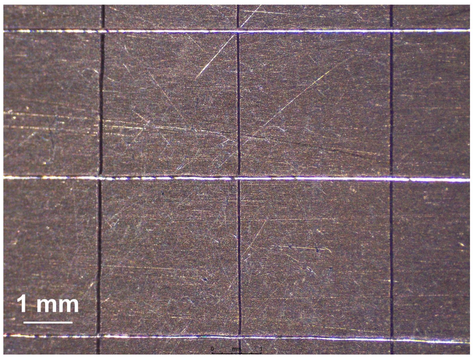

Figure S5. The adhesion of Zn@BP coatings on the surface of titanium alloys was examined using exfoliation experiments.

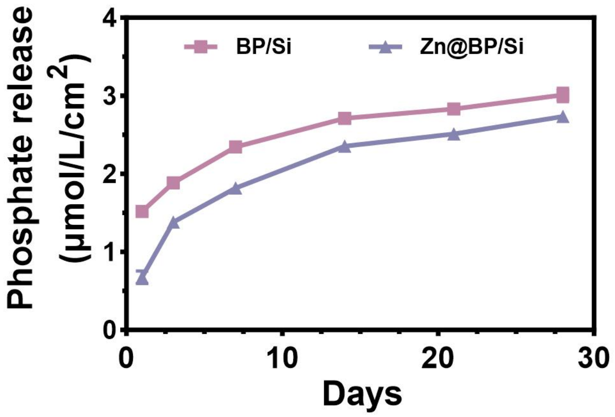

Figure S6. Phosphate released from Zn@BP/Si-coated 3D Ti scaffolds after different days of immersion in simulated body fluid solution under neutral conditions (pH=7.4).

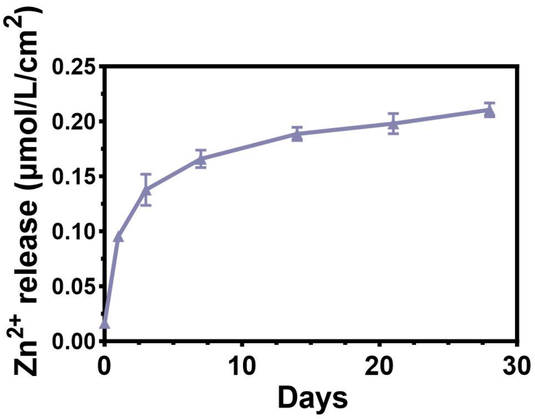

Figure S7. Zn^{2+} released from Zn@BP/Si-coated 3D Ti scaffolds after different days of immersion in simulated body fluid solution under neutral conditions (pH=7.4).

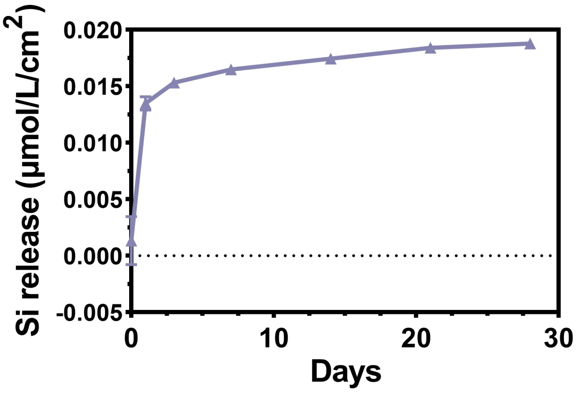

Figure S8. Si released from Zn@BP/Si-coated 3D Ti scaffolds after different days of immersion in simulated body fluid solution under neutral conditions (pH=7.4).

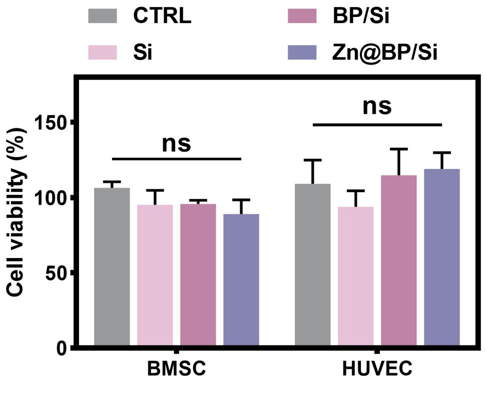

Figure S9. Relative cell viabilities of BMSCs and HUVECs cultured in medium containing different extracts at 3 days. *$P < 0.05$ compared with the DMEM group.

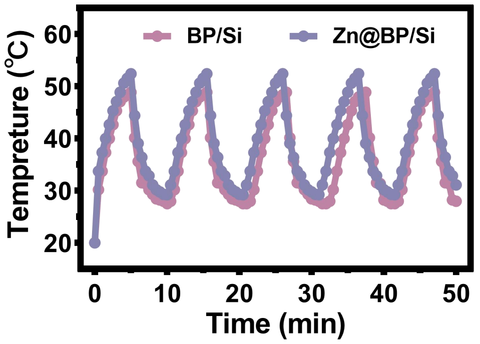

Figure S10. Photothermal stability properties of Zn@BP titanium alloy scaffolds in five laser switching cycles under 808 nm NIR laser.

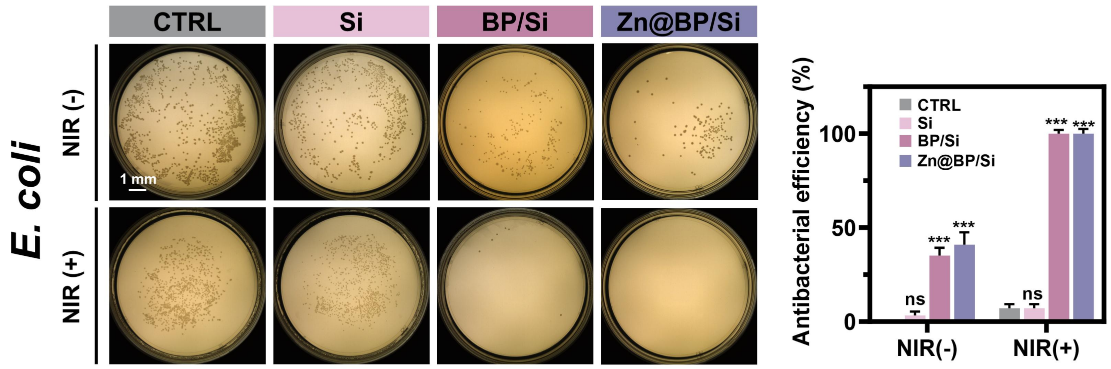

Figure S11. Colony culture and corresponding antimicrobial rates of *E. coli* under NIR irradiation or not.

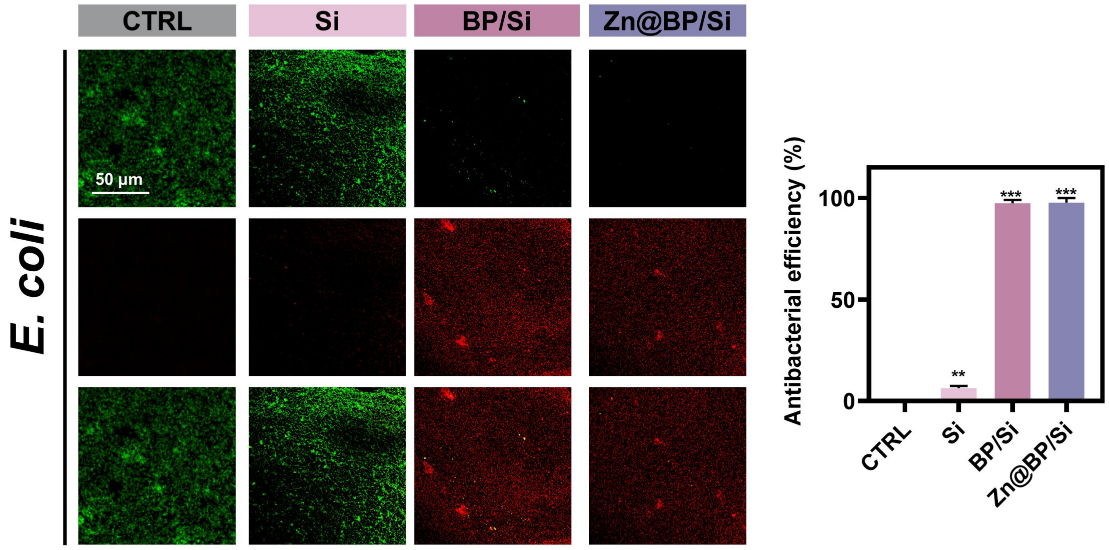

Figure S12. Live-dead bacterial staining of *E. coli* under NIR irradiation.

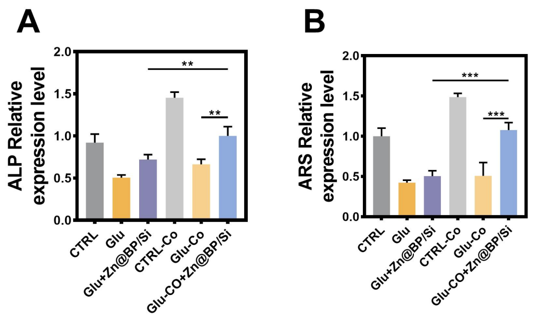

Figure S13. Relative quantitative analysis of BMSCs osteogenic differentiation on day 14 by ALP staining and the images (A) and relative quantitative analysis of BMSCs osteogenic differentiation on day 21 by the ARS staining (B). Data are present mean ± SD (n = 3). The mean was represented by columns and the SD were represented by error bars. **P* < 0.05, ***P* < 0.01, ****P* < 0.001.

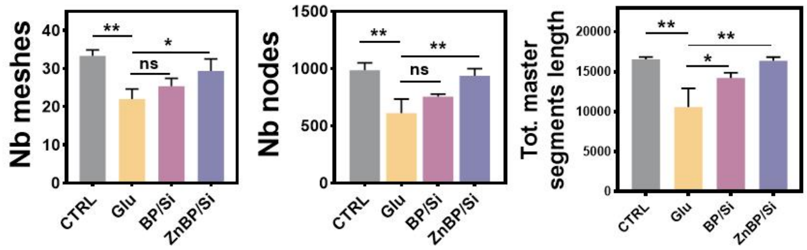

Figure S14. Histogram quantification of primary segment length (μm), number of nodes and grids of HUVECs after 6 h of culture in endothelial medium containing different coating extracts. Data are present mean ± SD (n = 3). The mean was represented by columns and the SD were represented by error bars. **P* < 0.05, ***P* < 0.01, ****P* < 0.001.

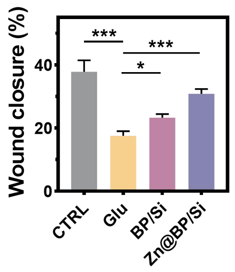

Figure S15. Percentage of wound healing after 24 h of culture with extracts. Data are present mean ± SD (n = 3). The mean was represented by columns and the SD were represented by error bars. **P* < 0.05, ***P* < 0.01, ****P* < 0.001.

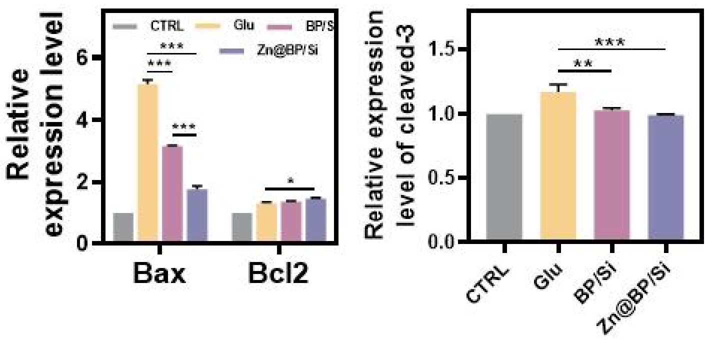

Figure S16. Quantitative analysis of Bax, Bal-2, caspase 3 and cleaved-3 expressions. $*P < 0.05$, $**P < 0.01$, $***P < 0.001$.

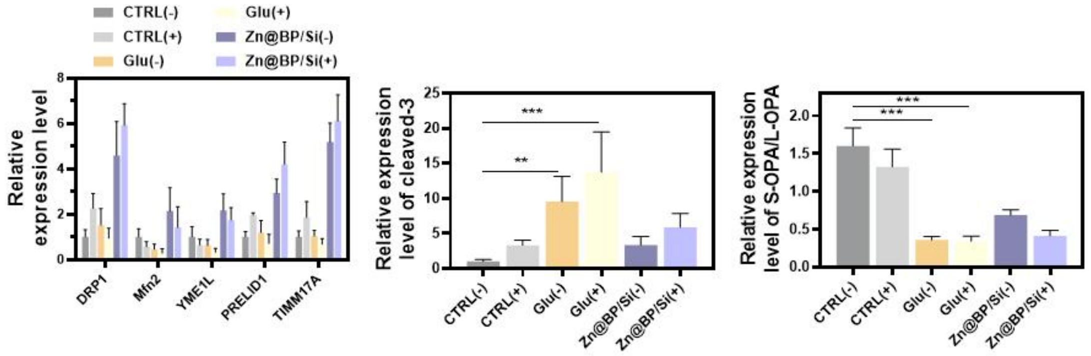

Figure S17. Quantitative analysis of Bax, Bal-2, caspase 3 and cleaved-3 expressions. $*P < 0.05$, $**P < 0.01$, $***P < 0.001$.

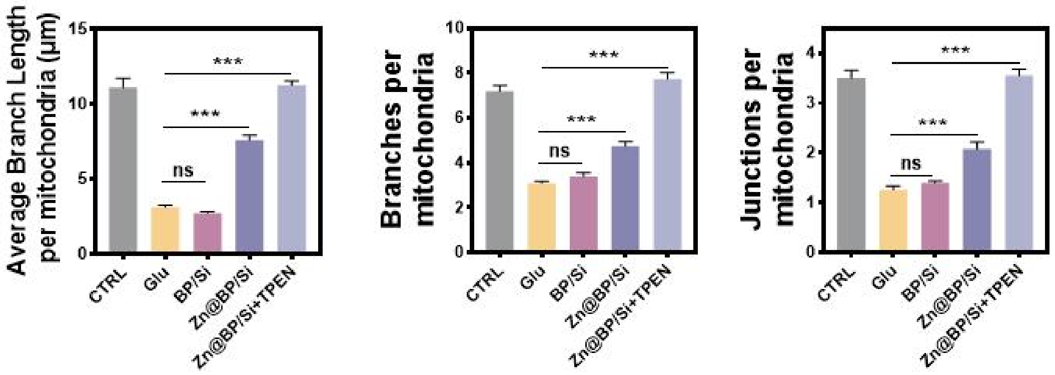

Figure S18. The classification was based on the area percentage of punctal, intermediate, and filamentous mitochondria under different sample groups (n = 10 cells), and the sphericity, width, and number of mitochondrial cristae were calculated by Image J. $*P < 0.05$, $**P < 0.01$, $***P < 0.001$.

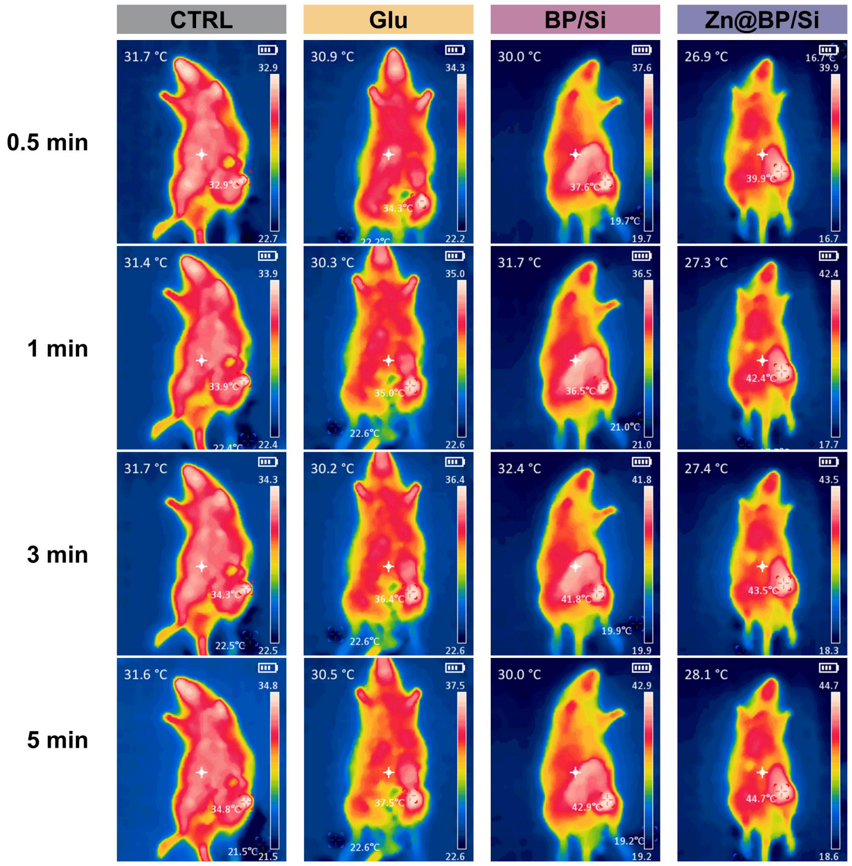

Figure S19. Photothermal imaging of diabetic femoral bone defect in rats with 3D-printed titanium alloy scaffolds with different coatings.

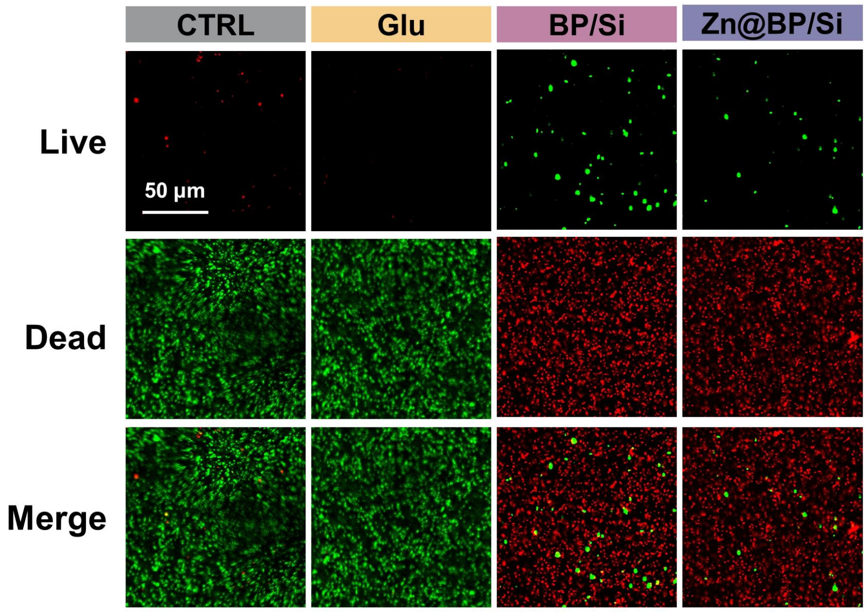

Figure S20. Images of *S. aureus* colonies from the surface of different implant samples.
